# Naringenin Inhibits Cellular Proliferation, Arrests Cell Cycle and Induces Pro‐Apoptotic Autophagy in MCF‐7 Breast Cancer Cells

**DOI:** 10.1111/jcmm.70747

**Published:** 2025-08-03

**Authors:** Suhail Ahmad Mir, Basharat Ahmad Bhat, Priti S. Shenoy, Laraibah Hamid, Nasir Nisar, Ashraf Dar, Pritha Ray, Ghulam Nabi Bader

**Affiliations:** ^1^ Department of Pharmaceutical Sciences University of Kashmir Srinagar Jammu and Kashmir India; ^2^ Department of Bio‐Resources, School of Biological Sciences University of Kashmir Srinagar Jammu and Kashmir India; ^3^ Imaging Cell Signaling and Therapeutics Lab Advanced Centre for Treatment Research and Education in Cancer, Tata Memorial Centre Navi Mumbai India; ^4^ Homi Bhabha National Institute BARC Training School Complex Anushaktinagar Mumbai India; ^5^ Department of Zoology University of Kashmir Srinagar Jammu and Kashmir India; ^6^ Department of Biochemistry University of Kashmir Srinagar Jammu and Kashmir India

**Keywords:** apoptosis, autophagy, breast cancer, cell cycle, naringenin

## Abstract

The global incidence of breast cancer has significantly increased, highlighting the need for novel therapeutic strategies. Current treatment options are often limited by drug resistance and adverse effects, necessitating the exploration of alternative compounds. Naringenin, a naturally occurring flavonoid in citrus fruits, exhibits antimicrobial, anti‐atherogenic, hepatoprotective, anti‐inflammatory, and anticancer properties. This study evaluates the potential of naringenin as an inhibitor of breast cancer cell proliferation. MCF‐7 breast cancer cells were used as a model system to assess the anti‐proliferative effects of naringenin. Cell viability was evaluated using MTT and colony formation assays, while cell migration was analysed via wound healing assay. Flow cytometry and western blotting were performed to examine cell cycle arrest and apoptosis, and autophagy was assessed through western blotting and confocal microscopy. Naringenin inhibited cellular proliferation in a dose‐dependent manner by arresting cells in the S‐phase of the cell cycle. It significantly reduced cellular migration and increased early and late apoptosis. Autophagy induction was confirmed by elevated LC3‐II expression, p62 degradation, and LC3‐II–LAMP1 co‐localization. Additionally, C‐PARP expression was reduced when cells were co‐treated with naringenin and 3‐methyladenine (3‐MA), indicating pro‐apoptotic autophagy. This study demonstrates the anti‐migratory and anti‐proliferative effects of naringenin and its ability to induce pro‐apoptotic autophagy in human breast cancer cells, suggesting its potential as a therapeutic agent.

**Abbreviations**
3‐MA: 3‐methyladenine
ACD: autophagic cell death
AMPK: AMP‐activated protein kinase
AO/EB: acridine orange/ethidium bromide
ATP: adenosine triphosphate
BBB: blood–brain barrier
BSA: bovine serum albumin
CDK: cyclin‐dependent kinase
C‐PARP: cleaved poly(ADP‐ribose) polymerase
DAPI: 4′,6‐diamidino‐2‐phenylindole
DCFDA: 2′,7′‐dichlorodihydrofluorescein diacetate
DMEM: Dulbecco's modified eagle medium
DMSO: dimethyl sulfoxide
DNA: deoxyribonucleic acid
ER: oestrogen receptor
FACS: fluorescence‐activated cell sorting
FBS: fetal bovine serum
FITC: fluorescein isothiocyanate
FSC: forward scatter
GAPDH: glyceraldehyde 3‐phosphate dehydrogenase
HER2: human epidermal growth factor receptor 2
HIF‐1α: hypoxia‐inducible factor 1‐alpha
IC_50_
: half maximal inhibitory concentration
LAMP‐1: lysosome‐associated membrane protein 1
LC3: microtubule‐associated protein 1 light chain 3
MAPK: mitogen‐activated protein kinase
MCF‐7: Michigan Cancer Foundation‐7 (breast cancer cell line)
MDA‐MB‐231: a triple‐negative breast cancer cell line
MFI: median fluorescence intensity
MMP: mitochondrial membrane potential
mTOR: mammalian target of rapamycin
MTT: 3‐(4,5‐dimethylthiazol‐2‐yl)‐2,5‐diphenyl tetrazolium bromide
NAC: N‐acetylcysteine
NAR: naringenin
NF‐κB: nuclear factor kappa B
Nrf2: nuclear factor erythroid 2‐related factor 2
p62: sequestosome‐1
PBS: phosphate‐buffered saline
PFA: paraformaldehyde
PI: propidium Iodide
PI3K: phosphoinositide 3‐kinase
PVDF: polyvinylidene difluoride
qRT‐PCR: quantitative real‐time polymerase chain reaction
ROS: reactive oxygen species
RPMI: Roswell Park Memorial Institute Medium
SDS‐PAGE: sodium dodecyl sulfate‐polyacrylamide gel electrophoresis
SSC: side scatter
TBST: tris‐buffered saline with tween
TEM: transmission electron microscopy

## 1 Introduction

Cancer is a global burden. As per the GLOBOCAN 2020 data, more than 19.3 million new cases and 10 million mortalities took place in the year 2020 due to this deadly disease. Notably, female breast cancer overtook lung cancer, emerging as the most frequently occurring cancer (constituting 11.7% of the total cases), followed by lung cancer (11.4%), colorectal cancer (10%), and prostate cancer (7.3%) [1, 2]. Globally, breast cancer ranks as the second most commonly diagnosed cancer [3, 4]. Worldwide, breast cancer is detected mostly in females [5] is the leading cause of death among them, primarily due to its propensity to metastasise at a faster rate, besides posing challenges in diagnosis and limited available therapeutic options [6]. Breast cancer in India accounts for 7% of all breast cancer cases worldwide and one‐fifth of all female malignancies [7]. According to epidemiological studies, the global incidence of breast cancer is projected to surpass 2 million cases by the year 2030 [8]. Radiation therapy, mammography, chemotherapy, hormone therapy, and surgery are the most frequently employed therapeutic approaches in the diagnosis and management of breast cancer [9, 10, 11]. The effectiveness of widely used chemotherapeutic agents is continually on a decline owing to the development of resistance, recurrence of the disease, and occurrence of adverse side effects [12, 13]. Multidrug resistance (MDR) presently is a profound concern inherent in the existing diagnostic approach [14] as due to this, the survival rate of individuals diagnosed with breast cancer has significantly declined. Therefore, novel therapeutic approaches that can inhibit metastasis and cellular migration and enhance clinical outcomes in breast cancer patients are extremely important [15]. Natural products from plants, animals, or minerals exhibit various biological activities [16]. Bioactive molecules from medicinal plants have shown a significant therapeutic potential in cancer chemoprevention [17, 18]. Many studies have reported that flavonoids isolated from citrus fruits, vegetables, and dietary substances possess significant anticancer potential [19, 20].

Among the various potent natural anticancer molecules, Naringenin (NAR), chemically known as 5,7‐dihydroxy‐2‐(4‐hydroxyphenyl) 2,3‐dihydrochromen‐4‐one, is a hydrophobic citrus flavonone (type of flavonoid), belonging to the family Vitamin P [21]. It has been found to induce apoptosis and cause cell cycle arrest in a number of studies that used various cancer cell lines, including the MDA‐MB‐231 cell line [22]. Lee et al. in their study, observed that overproduction of reactive oxygen species (ROS) by NAR results in activation of endoplasmic reticulum stress, which leads to induction of autophagy and apoptosis in human osteosarcoma cells [23]. In a similar study, Shi et al. observed that NAR inhibits cellular migration, causes cell cycle arrest, and induces apoptosis in human lung cancer, A549 cell line [24]. Naringin, whose aglycone is NAR, has been found to induce antiproliferative activity in human colorectal cancer (HCT‐116 cell line) by suppressing the PI3K/AKT/mTOR signalling cascade [25]. Despite growing evidence of NAR's anticancer potential, its precise mechanistic role in breast cancer remains incompletely understood. While previous studies have demonstrated its ability to induce apoptosis, inhibit migration, and regulate autophagy in various cancer models, the interplay between these processes, particularly the crosstalk between apoptosis and autophagy in breast cancer cells, remains unclear. Additionally, the role of ROS generation as a potential upstream regulator of these pathways has not been thoroughly explored. This study aims to address these gaps by systematically investigating the effects of NAR on breast cancer cell proliferation, apoptosis, and autophagy, with a specific focus on ROS‐mediated mechanisms. By integrating multiple in vitro assays, including cell viability, migration, flow cytometry, western blotting, and fluorescence microscopy, this research provides a comprehensive mechanistic evaluation of NAR's therapeutic potential in breast cancer. Furthermore, this study explores the effect of 3‐methyladenine (3‐MA), an autophagy inhibitor, to determine whether NAR‐induced cell death is primarily apoptotic or autophagy‐dependent. These findings will contribute to a deeper understanding of how NAR modulates cancer cell fate and may offer insights for its potential use in breast cancer therapy.

## 2 Materials and Methods

### 2.1 Materials

NAR (Sigma, N5893), fetal bovine serum/FBS (gibco/Invitrogen, USA), DMSO (sigma USA, D2438), RPMI‐1640 (gibco, 2541822), streptomycin/penicillin (gibco/Invitrogen, USA), PI (sigma, USA, P4864), MTT (Sigma, 298931), trypsin–EDTA (gibco/Invitrogen, USA), 3‐MA, DCFDA (ABCAM, ab113851), cisplatin, DAPI (Sigma, D9542), RNase A (Sigma USA, 9001‐99‐4), methyl blue, LC3‐II (CST, 2775S), P62 (Protein‐Tech, 18420–1‐AP, rabbit), LAMP1 (monoclonal antibody, H4A3) C‐PARP (CST, D2149541S, rabbit), Annexin‐V Fluor 488 (Thermofisher Scientific, V13241) were used in the study. All reagents were of analytical grade and used according to the manufacturer's instructions.

### 2.2 Cell Line and Culture

MCF‐7 breast cancer cells (ATCC, USA) were cultured in RPMI‐1640 medium supplemented with 10% FBS and 1% penicillin–streptomycin. Cells were maintained in a 5% CO_2_ incubator at 37°C under humidified conditions.

### 2.3 MTT Assay

The antiproliferative activity of NAR was determined by 3‐(4,5‐dimethylthiazol‐2‐yl)‐2,5‐diphenyltetrazolium bromide (MTT) assay, following the method of Wang et al. with slight modifications [26]. MCF‐7 cells were seeded at a density of 1 × 10^4^ cells/100 μL per well in a 96‐well plate and incubated for 24 h at 37°C in a 5% CO₂ atmosphere. Cells were then treated with different concentrations of NAR (dissolved in DMSO as a vehicle control) for 24 and 48 h. After treatment, 10 μL of MTT solution (5 mg/mL in PBS) was added to each well and incubated for 4 h in a CO₂ incubator. The supernatant was carefully removed, and 100 μL of DMSO was added to dissolve the formazan crystals. Absorbance was measured at 570 nm using a Bio‐Rad microplate reader, and IC_50_ values were calculated.

### 2.4 Morphological Assay for Apoptosis

To assess the apoptotic effects of NAR, we followed the method described by Ajji et al. with slight modifications [27]. MCF‐7 cells were treated with increasing concentrations of NAR (50, 100, and 150 μM) for 24 h, after which cellular morphology was examined using a Nikon Eclipse Ti fluorescence microscope (USA) at 10× magnification. Images were captured before and after treatment to analyse apoptotic changes.

### 2.5 Wound Healing Assay

The wound healing assay was performed following the method described by Wang et al. with slight modifications [28]. MCF‐7 cells (1.5 × 10^6^ cells/well) were seeded in a six‐well plate and cultured until 90% confluency. A uniform scratch was created using a sterile 200 μL pipette tip, and detached cells were removed by washing with phosphate‐buffered saline (PBS). Cells were then treated with various concentrations of NAR (50, 100, and 150 μM), with cisplatin (20 μg/mL) as a positive control, for 24 h. Scratch closure was monitored, and images were acquired at 0, 24, 48, and 72 h using a Nikon Eclipse Ti microscope (USA) with an Andor Clara DR‐4285 TI imaging system at 10× magnification. Quantitative analysis of wound closure was performed using ImageJ software, and migration rates were calculated using the standard formula [29].

### 2.6 Colony Formation Assay

The colony formation assay was conducted as described by Franken et al. with slight modifications [30]. MCF‐7 cells were harvested, counted, and seeded into six‐well plates at a density of 300 cells per well. After 24 h, cells were treated with NAR (1, 10, 20, 40, and 50 μM) for another 24 h. Subsequently, the medium was replaced with fresh drug‐free culture medium, and cells were allowed to grow for an additional 10 days. Colonies were then fixed with methanol for 15 min, stained with 0.5% crystal violet for 15 min, and washed with PBS. Colonies containing ≥ 50 cells were counted, and colony survival rates were determined as a percentage relative to the untreated control.

### 2.7 Cell Cycle Analysis by Propidium Iodide Staining

Cell cycle distribution was analysed following the method of Xu et al. with slight modifications [31]. MCF‐7 cells (1.5 × 10^6^ cells/well) were seeded in six‐well plates and cultured until they reached approximately 70% confluency. Cells were then treated with NAR (50, 100, and 150 μM) for 24 h, after which they were trypsinised, harvested, and centrifuged at 1000 rpm for 4 min at 4°C. The cell pellet was fixed by adding 1 mL of ice‐cold 70% ethanol dropwise with gentle vortexing, followed by incubation on ice for 30 min. Fixed cells were centrifuged at 1200 rpm for 5 min, washed twice with 1× PBS to remove residual ethanol, and re‐suspended in 250 μL of PI staining buffer (1× PBS containing 1 mg/mL PI and 10 μg/mL RNase A). Samples were incubated at 37°C in the dark for 15 min with intermittent tapping to prevent clumping. Cell cycle analysis was performed using an Attune NxT acoustic focusing cytometer (USA), and data were analysed with FlowJo VX version 10.1 software.

### 2.8 Acridine Orange and Ethidium Bromide Staining for Apoptosis

Apoptotic cell death was assessed using Acridine Orange and Ethidium Bromide (AO/EB) dual staining as described by Alfaifi with slight modifications [32]. MCF‐7 cells (1.5 × 10^6^ cells/well) were seeded in six‐well plates and treated with increasing concentrations of NAR (50, 100, and 150 μM) for 24 h. Following treatment, cells were trypsinised with 0.25% trypsin–EDTA, washed twice with 1× PBS, and re‐suspended in PBS. A 20 μL aliquot of the cell suspension was mixed with 2 μL of AO/EB solution (100 μg/mL of acridine orange and 100 μg/mL of ethidium bromide in 1× PBS) on a glass slide. Cells were immediately visualised under a Nikon Eclipse E400 fluorescence microscope (USA) to distinguish live, early apoptotic, late apoptotic, and necrotic cells based on nuclear morphology and fluorescence emission.

### 2.9 DAPI Staining for Apoptosis

Cells were seeded in a 6‐well plate at a density of 1.5 × 10^6^ cells/well, following the method of Xie et al. with slight modifications [33]. After achieving the desired confluency, the cells were treated with different concentrations of NAR (50, 100, and 150 μM) for 24 h. Cells were then washed twice with 1X PBS to remove any cell residue and fixed with 4% paraformaldehyde for 10 min at 37°C. After fixing, the cells were permeabilised with permeabilising buffer (3% PFA and 0.5% Triton X‐100) and subsequently stained with 4′, 6‐diamino‐2‐phenylindole dihydrochloride (DAPI) (1 mg/mL) dye for 30 s. Images were taken to observe any nuclear changes using a fluorescent microscope, LSM760 confocal, from the USA.

### 2.10 Annexin V/PI Staining by Flowcytometry

Cheng et al.'s method using Annexin‐V/PI double staining with slight modifications [25] was used for this. Cells were seeded in a 6‐well plate at a density of 1.5 × 10^6^ cells/well and treated with different concentrations of NAR (50, 100, and 150 μM) for 24 h. Cells were then harvested by trypsinisation and centrifuged at 1000 rpm for 5 min. After this, cells were washed twice with 1× PBS and centrifuged at 1500 rpm for 5 min. Cells were then suspended in 100 μL of 1× Annexin binding buffer, and to each FACS tube, 1.5 μL of Annexin V was added and kept in the dark for 15 min at room temperature. After 15 min, 1 μL of PI was added to each FACS tube and analysed by flow cytometry. Attune NxT acoustic focusing cytometer–USA, using FlowJo VX version 10.1 software was used for the purpose. Annexin V used was conjugated to Alexa Fluor 488, with excitation/emission (499, 535/521, 617).

### 2.11 Intracellular Reactive Oxygen Species Determination by Flowcytometry

Zhang et al.'s method with slight modifications [34] was used for reactive oxygen species (ROS) determination. Cells were trypsinised after treatment with different concentrations of NAR (50 and 100 μM) for 24 h. Cells were centrifuged at 1000 rpm for 5 min, and the pellet was dissolved in 200 μL of 0.5 μM 2′,7‐dichlorodihydrofluorecein diacetate (DCFDA) dye (5 mM, in DMSO) and kept in an incubator wrapped in aluminium foil for 25 min at 37°C. Finally, ROS generation was calculated as median fluorescence intensity (MFI) by flow cytometry, Attune NxT acoustic focusing cytometer‐USA, using FlowJo VX version 10.1 software. The stock solution of DCFDA had excitation/emission spectra of 495 and 529 nm, respectively.

### 2.12 Autophagy Through Immunofluorescence

Xiong et al.'s method with slight modifications [35] was used for the assay. Proliferating MCF‐7 cells were allowed to grow on cover slips in a 6‐well plate until 70% confluency was achieved. MCF‐7 cells were then treated with different concentrations of NAR (50, 100, and 150 μM) for 24 h. Media was aspirated and cells were fixed with 1 mL 4% paraformaldehyde (PFA) and incubated for 10 min at 37°C. PFA was aspirated, and cells were given three gentle washings with 1× PBS, followed by blocking with 3% bovine serum albumin (BSA) on a shaker at low speed for 30 min. Cover slips were placed (upside down) on 50 μL of primary antibody (LC3‐II and LAMP1 in the ratio of 1:100 in 3% BSA) on a paraffin film (prepared as per the lab protocol) in such a way that cells faced the antibody and were kept at 4°C for 24 h. The next day, cells were removed from the refrigerator and given 3 gentle washings with 1X PBS. Cells were then put into 50 μL secondary antibody (LC3‐488‐Green‐Rabbit and LAMP1‐633‐Red‐Mouse in the ratio of 1:200 in PBS), in a similar manner as primary antibody for 2 h in the dark. After removal from the secondary antibody, cells were again given 3 gentle washings with 1X PBS before putting them into DAPI (1:3000 in 1X PBS) for 5 s and were again given 3 gentle washings with 1X PBS. On a glass slide, a drop of Vectashield was put, and cover slips were placed on the glass slide with cells facing toward the Vectashield. Edges of cover slips were then fixed with transparent nail polish (as per lab protocol). After 5–10 min, slides were analysed under a Nikon AX confocal microscope, USA, for puncta formation. Nikon NIS Element was used for image acquisition.

### 2.13 Western Blotting

Western blotting was done by the method of Ahsan et al. with slight modifications [36]. MCF‐7 cells were treated with different concentrations of NAR (50, 100, and 150 μM) for 24 h. Cell lysate was collected by plate lysing, using radioimmunoprecipitation assay buffer (RIPA), (5 nM EDTA, 1 mM Na3V04, 1% protease inhibitor cocktail, 1 mM PMSF, 150 mM NaCl, and 20 mM Tris–HCl) for 45 min on ice. Protein estimation was done using the Bradford assay. A protein mixture of 40 and 60 μg for LC3‐II, P62, and C‐PARP was loaded in a 12%, and 8% gel separately. The gel was allowed to run at 60 V, 400 mA, and gradually the voltage was increased to 80 V. The gel was then transferred to a PVDF membrane into a transfer tank for about 1–2 h at 13 V, 400 mA. After complete transfer, the PVDF membrane containing proteins was subjected to blocking with 3% BSA for 1 h at room temperature, followed by incubation with primary antibody LC3 (1:1000), P62 (1:5000), C‐PARP (1:1000) for 24 h at 4°C with gentle shaking. After incubation with primary antibody, the membrane was given 3 washings of 5 min each with wash buffer (1×TBSt) on a plate shaker and incubated with secondary antibodies (GAPDH and α‐tubulin) for about 2 h. Proteins were visualised by Chemidoc, Bio‐Rad enhanced chemiluminescence (ECL).

### 2.14 Statistical Analysis

One‐way ANOVA and Student's *t*‐test were used for the analysis using GraphPad Prism V8.0.2 software. Data is expressed as mean ± SD. All the experiments were carried out in triplicate to ensure reproducibility.

## 3 Results

**FIGURE 1:**
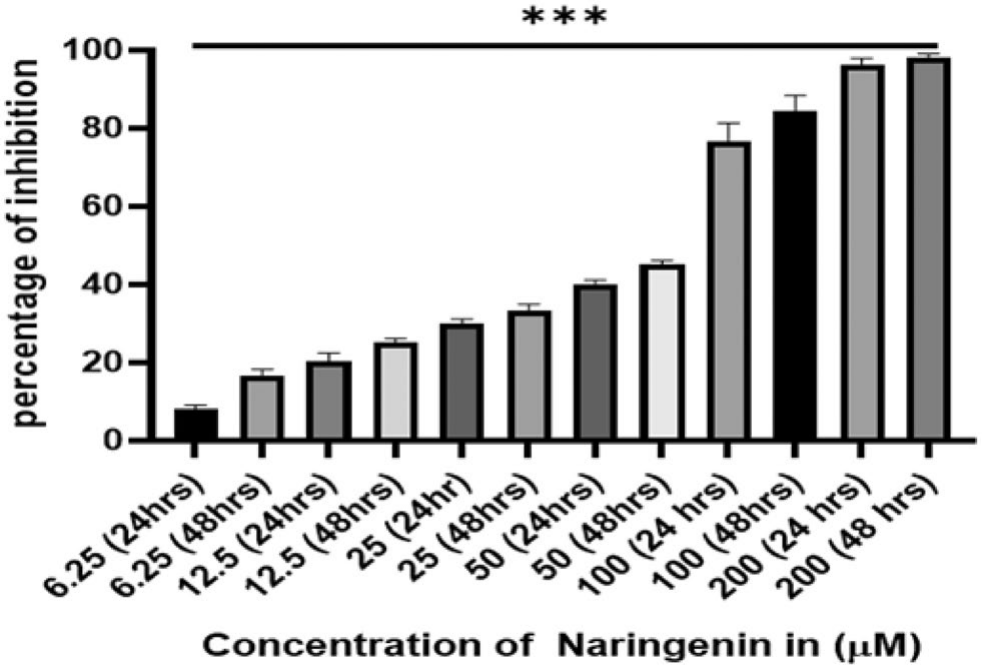
Antiproliferative potential of naringenin against human breast cancer MCF‐7 cells, as assessed by the MTT assay. The percentage of cell viability inhibition was found to be both time‐ and dose‐dependent. Naringenin exhibited an IC₅₀ of 95 μM after 24 h of treatment and 49 μM after 48 h, indicating enhanced cytotoxicity with prolonged exposure. Statistical significance is denoted by ***p < 0.0001. Data are presented as mean ± SD from three independent experiments.

We first investigated the effect of NAR on cellular proliferation against the MCF‐7 cell line using 3‐(4,5‐dimethylthiazol‐2yl)‐2,5‐diphenyltetrazolium bromide (MTT) assay. The assay revealed a dose‐and time‐dependent decrease in cell viability, indicating enhanced cytotoxicity with prolonged exposure to NAR (Figure 1), thereby revealing its strong anti‐proliferative potential, and accordingly IC_50_ was calculated.

**FIGURE 2:**
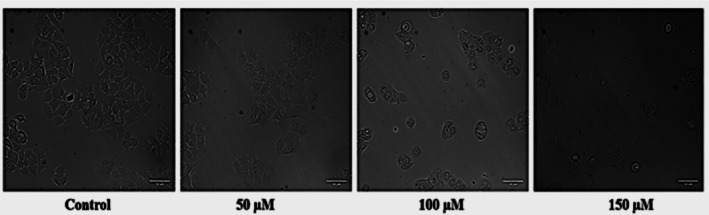
Effect of Naringenin on MCF‐7 cell morphology in a dose‐dependent manner. Representative phase‐contrast images showing morphological changes in MCF‐7 breast cancer cells after 24 h of treatment with increasing concentrations of naringenin (50, 100, and 150 μM). Control cells exhibit normal morphology with high confluency, whereas naringenin‐treated cells display a concentration‐dependent decrease in cell density, increased rounding, and detachment, indicative of apoptosis.

We next investigated the morphological changes associated with the use of naringenin against the MCF‐7 cell line. The findings revealed that cells treated with NAR exhibited more pronounced apoptotic morphological changes, such as cell shrinkage, loss of cell connections, membrane disintegration, and an increased number of dead cells, compared to the control (Figure 2).

**FIGURE 3:**
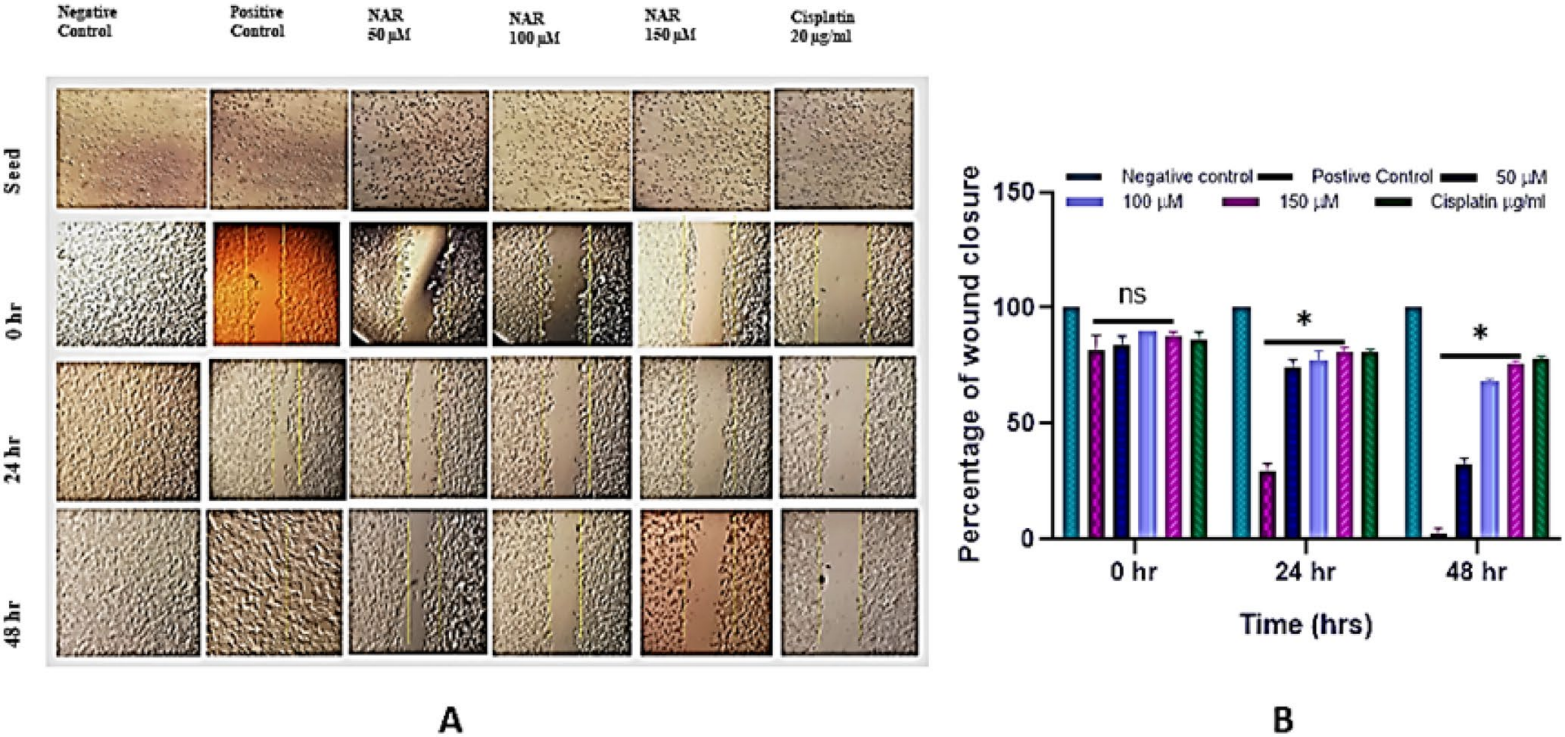
Effect of Naringenin on MCF‐7 cell migration assessed by wound healing assay. (A) Representative phase‐contrast images showing the effect of naringenin (NAR) at different concentrations (50, 100, and 150 μM) on wound closure in MCF‐7 cells over 48 h. Images were taken at 0, 24, and 48 h post‐scratch. Negative control represents untreated cells, and cisplatin (20 μg/mL) served as a positive control. (B) Quantification of wound closure percentage over time. Data represent mean ± SD from three independent experiments. Statistical significance is indicated (*p < 0.05).

We further investigated the effect of NAR on cellular migration using a wound healing assay. A wound was created by holding a sterile micropipette tip perpendicular to the plate. Detached cells were removed by washing with 1X PBS, followed by treatment with varying concentrations of NAR (50, 100, and 150 μM). Cisplatin (20 μg/mL) was used as a positive control. The assay demonstrated a concentration decrease in cellular migration with increasing concentrations of naringenin (Figure 3). Migration was calculated in the cell‐free region.

**FIGURE 4:**
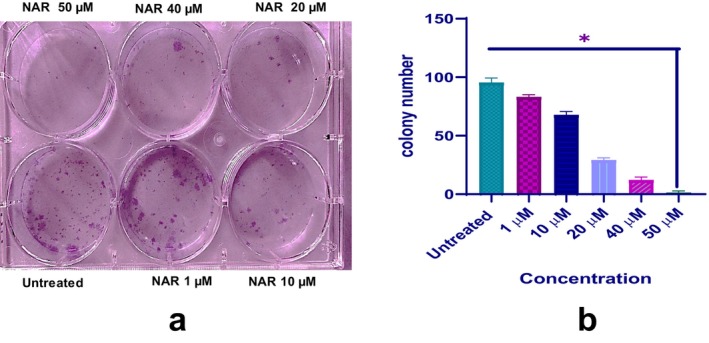
Effect of Naringenin on MCF‐7 cell proliferation evaluated by colony formation assay. (a) Representative image of a colony formation assay showing MCF‐7 cell growth after treatment with increasing concentrations of naringenin (1, 10, 20, 40, and 50 μM) for 10 days. Cells were stained with crystal violet. (b) Quantification of colony formation. Data represent mean ± SD from three independent experiments. Statistical significance is indicated (*p < 0.05).

We examined the effect of NAR on colony formation, and the results revealed a significant, dose‐dependent reduction in colony count compared to the control (Figure 4).

**FIGURE 5:**
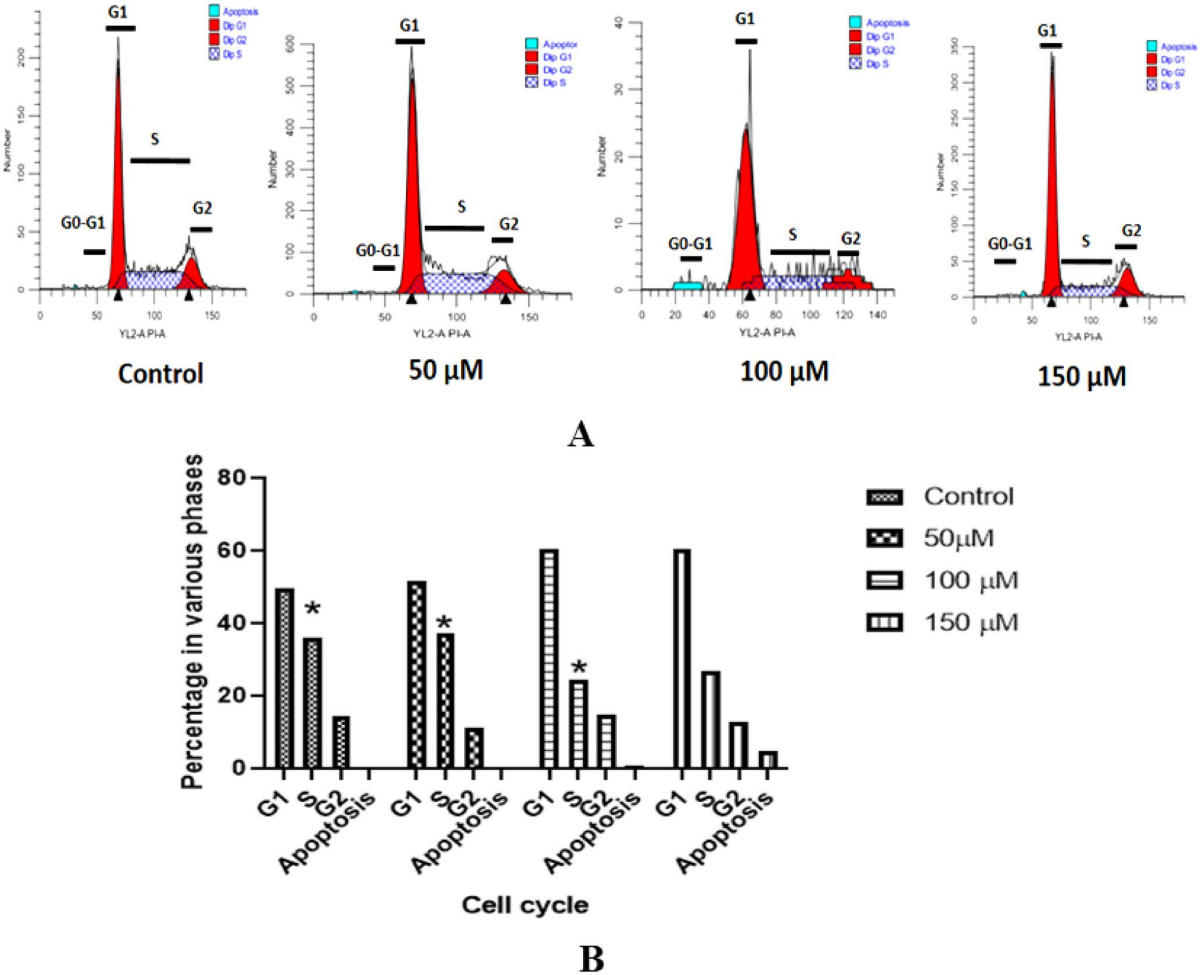
(A) Flow cytometry histograms showing cell cycle distribution in MCF‐7 cells treated with increasing concentrations of NAR (50, 100, and 150 μM) for 24 h. Cells were stained with propidium iodide (PI) and analysed using an Attune NxT acoustic focusing cytometer. The G0/G1, S, and G2 phases are marked, along with a sub‐G1 apoptotic population. (B) Quantification of cell cycle phase distribution. Data represent mean ± SD from three independent experiments. Statistical significance is indicated (*p < 0.05).

Next, we investigated the effect of NAR on the cell cycle (Figure 5). Flow cytometric analysis revealed that NAR induced cell cycle arrest at the G0/G,1 and S phase in a dose‐dependent manner in MCF‐7 cells. The percentage of cells in the G0/G1 phase increased from 49% to 61%, while the S phase population decreased from 37% to 26%.

**FIGURE 6:**
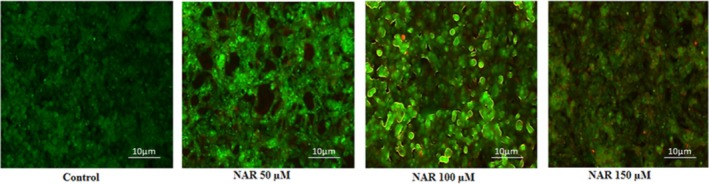
Apoptotic morphological changes induced by Naringenin in MCF‐7 cells assessed by AO/EB double staining. Representative fluorescence microscopy images of MCF‐7 cells stained with acridine orange (AO) and ethidium bromide (EB) after 24‐hour treatment with increasing concentrations of naringenin (50, 100, and 150 μM). Green fluorescence represents live cells, yellow‐green cells indicate early apoptosis with chromatin condensation, and red fluorescence denotes late apoptotic or necrotic cells. The increase in red fluorescence with higher NAR concentrations indicates dose‐dependent apoptosis.

We further investigated whether NAR could induce apoptosis using an acridine orange and ethidium bromide (AO/EB) double staining assay. The results confirmed that NAR induces both early and late apoptosis. Early apoptotic cells exhibited crescent‐shaped, yellowish green nuclei stained by acridine orange, while late apoptotic cells showed condensed, asymmetrically localised orange nuclei stained by ethidium bromide. In contrast, normal cells displayed intact nuclei with uniform central distribution (Figure 6).

**FIGURE 7:**
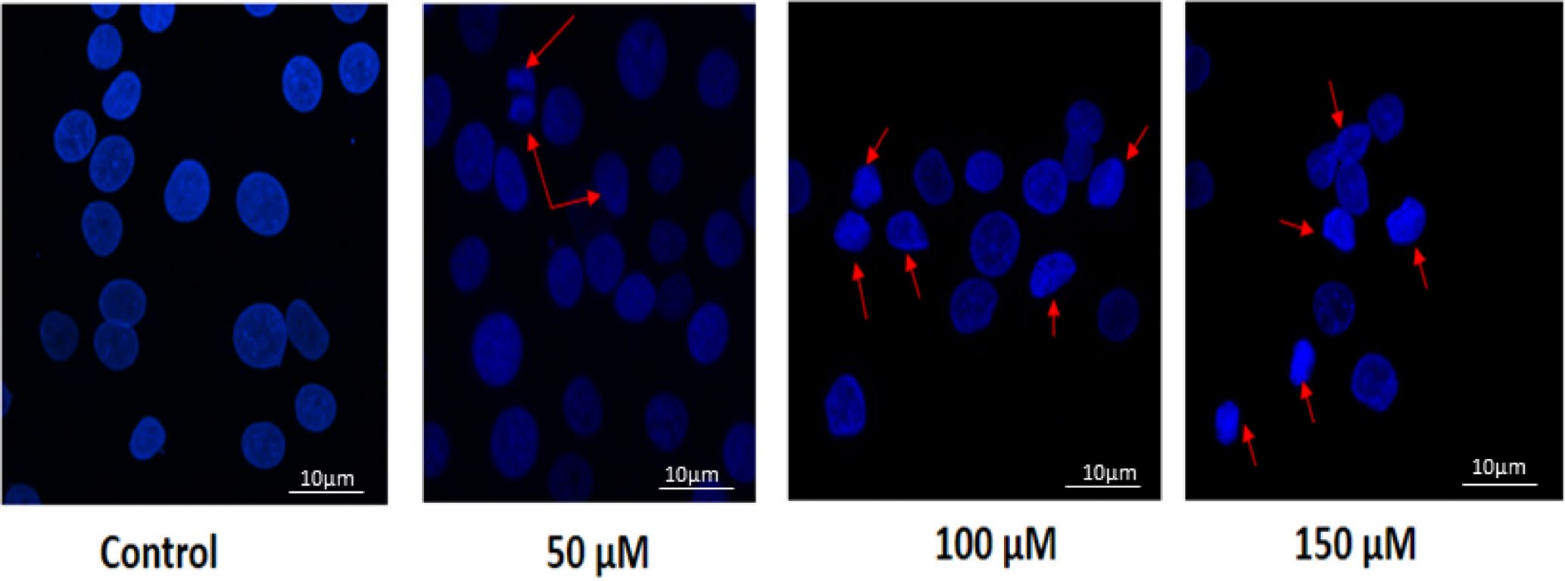
Naringenin‐induced chromatin condensation in MCF‐7 cells assessed by DAPI staining. Representative fluorescence microscopy images showing chromatin condensation in MCF‐7 cells treated with increasing concentrations of naringenin (50, 100, and 150 μM) for 24 h. Cells were stained with DAPI to visualise nuclear morphology. Control cells exhibit intact nuclei, whereas naringenin‐treated cells show dose‐dependent chromatin condensation and nuclear fragmentation (indicated by red arrows), characteristic of apoptosis. Images were taken using a fluorescent microscope, LSM760 confocal, USA.

To further evaluate its role in inducing apoptosis, we performed DAPI staining to assess naringenin‐induced chromatin condensation, a hallmark of apoptosis. The results revealed that NAR induces chromatin condensation as indicated by red arrows (Figure 7) in a dose‐dependent manner with maximum chromatin condensation achieved at 150 μM.

**FIGURE 8:**
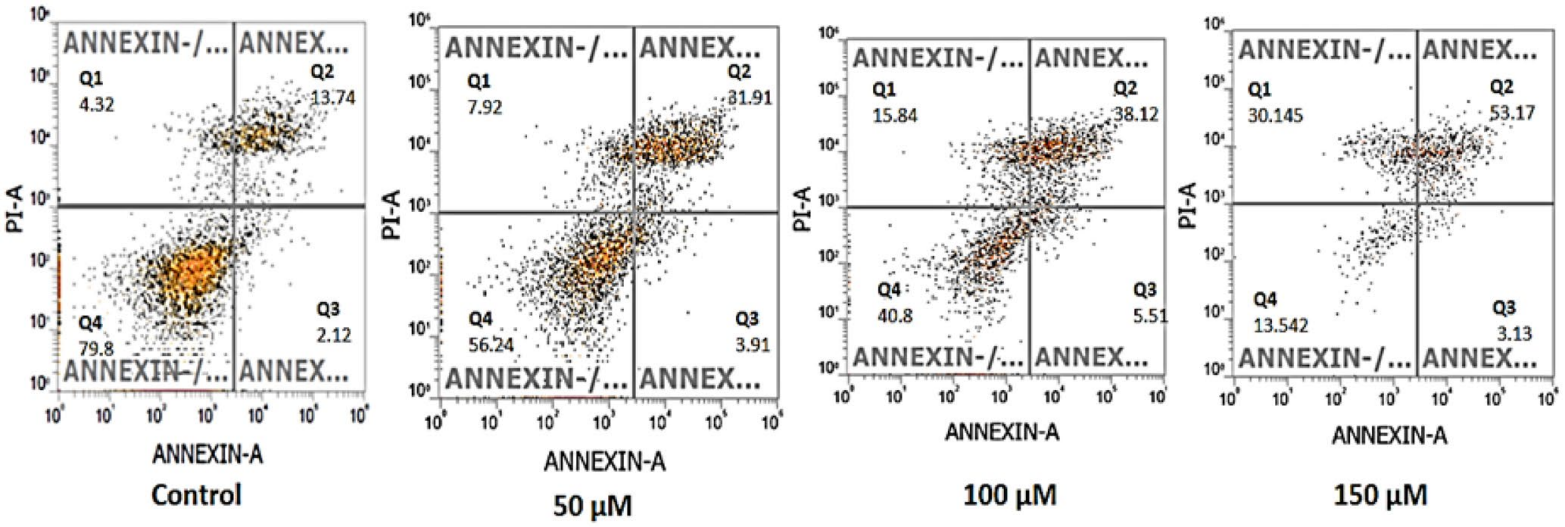
Annexin‐V/PI flow cytometry analysis of naringenin‐induced apoptosis in MCF‐7 cells. Flow cytometry dot plots representing Annexin‐V/PI staining in MCF‐7 cells treated with increasing concentrations of naringenin (50, 100, and 150 μM) for 24 h. Quadrants: Q1 (Top Left): Necrotic cells (Annexin‐V^−^/PI^+^); Q2 (Top Right): Late apoptotic cells (Annexin‐V^+^/PI^+^); Q3 (Bottom Right): Early apoptotic cells (Annexin‐V^+^/PI^−^); Q4 (Bottom Left): Live cells (Annexin‐V^−^/PI^−^). Apoptotic cell populations (Q2 and Q3) increase in a dose‐dependent manner. Data represent mean ± SD from three independent experiments. *p < 0.0001.

After confirming the pro‐apoptotic effect of NAR, we further evaluated the distribution of early apoptotic, late apoptotic, necrotic, and live cells in NAR‐treated MCF‐7 cells using Annexin‐V/PI double staining. The results revealed a concentration‐dependent increase in the percentage of early, late, and necrotic cells, accompanied by a corresponding decrease in the live cell population. The maximum effect was observed at 150 μM. Compared to the control group, the early apoptotic cell population increased from 2.12% to 3.13%, late apoptotic cells from 13.74% to 53.17%, and necrotic cells from 4.32% to 30.145%, while the live cell population decreased significantly from 79.8% to 13.542% (Figure 8).

**FIGURE 9:**
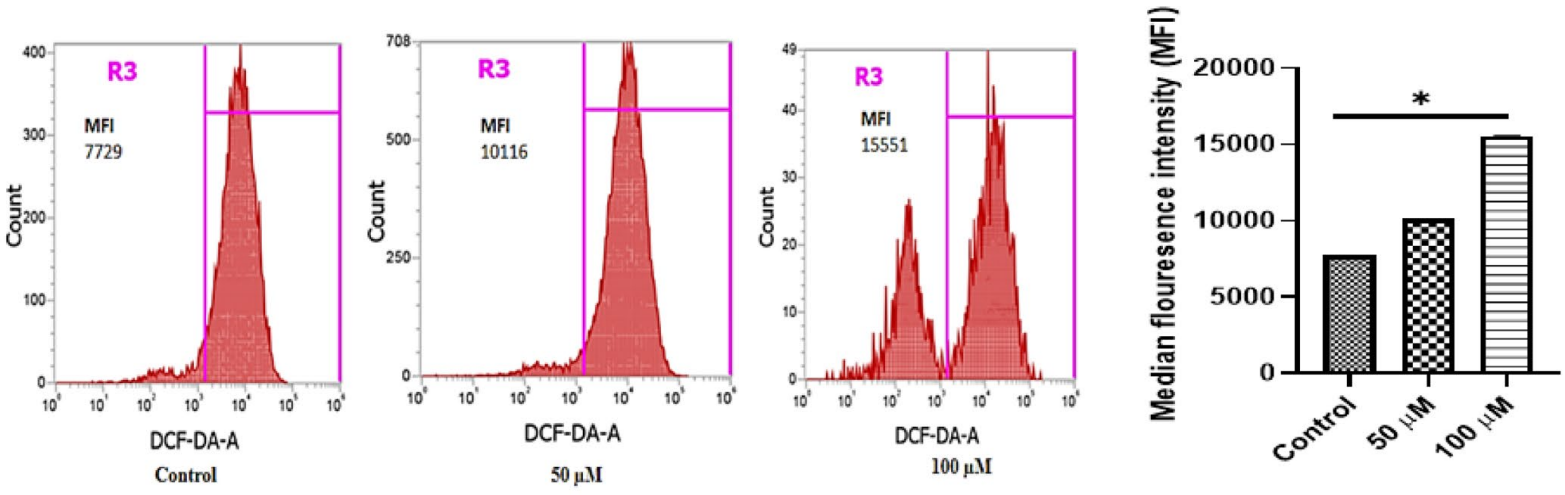
Naringenin‐induced ROS generation in MCF‐7 cells assessed by DCFDA staining and flow cytometry. Flow cytometry histograms representing ROS production in MCF‐7 cells treated with increasing concentrations of naringenin (50 and 100 μM) for 24 h. Cells were stained with 0.5 μM DCFDA for 25 min before analysis. ROS levels were quantified as median fluorescence intensity (MFI) in region R3 (highlighted in pink). (Left to right) Control (MFI = 7729), 50 μM NAR (MFI = 10,116), and 100 μM NAR (MFI = 15,551). The bar graph on the right quantifies MFI across conditions. Data represent mean ± SD from three independent experiments. *p < 0.05.

Next, we investigated whether NAR could induce ROS‐mediated apoptosis using the DCFH‐DA assay. NAR significantly increased the ROS generation, as evidenced by a concentration‐dependent rise in median fluorescence intensity (MFI) compared to the control. The highest ROS generation was observed at 100 μM, with a MFI value of 15,551 (Figure 9).

**FIGURE 10:**
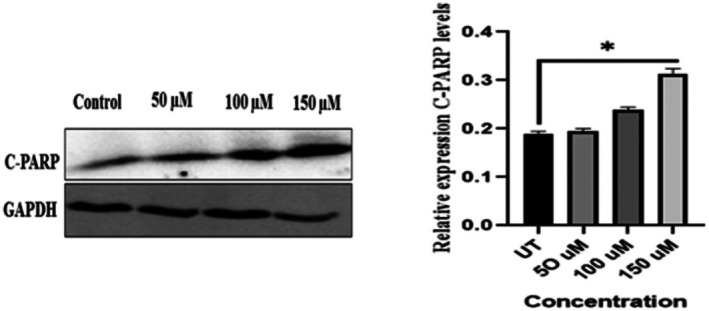
Western blot analysis of cleaved PARP expression in MCF‐7 cells treated with naringenin. (Left) Representative western blot showing cleaved PARP (C‐PARP) expression in MCF‐7 cells treated with different concentrations of naringenin (50, 100, 150 μM) for 24 h. GAPDH was used as a loading control. (Right) Quantification of relative C‐PARP expression normalised to GAPDH. Data represent mean ± SD from three independent experiments. Statistical significance is indicated (*p < 0.05).

To elucidate the apoptotic pathway activated by NAR, we performed western blot analysis to assess the expression of cleaved PARP (C‐PARP), a well‐established marker of programmed cell death. Our results demonstrated a significant dose‐dependent up‐regulation of C‐PARP expression in NAR‐treated MCF‐7 cells compared to the untreated control, confirming the induction of apoptosis (Figure 10).

To investigate whether NAR induces autophagy, we performed immunofluorescence staining using confocal microscopy to assess LC3‐II and LAMP‐1 puncta formation and their co‐localization, alongside western blot analysis of LC3‐II and p62 degradation. MCF‐7 cells were treated with NAR (50, 100, and 150 μM) for 24 h. For immunofluorescence analysis, cells were stained and visualised as described in the methodology, while western blotting was performed on cell lysates incubated with primary and secondary antibodies, as detailed in the methodology section. The results showed that NAR significantly increased LC3‐II and LAMP‐1 puncta formation and enhanced their co‐localization, indicating autophagosome–lysosome fusion. Moreover, NAR induced a dose‐dependent up‐regulation of LC3‐II protein expression and p62 degradation, confirming activation of autophagy.

**FIGURE 11:**
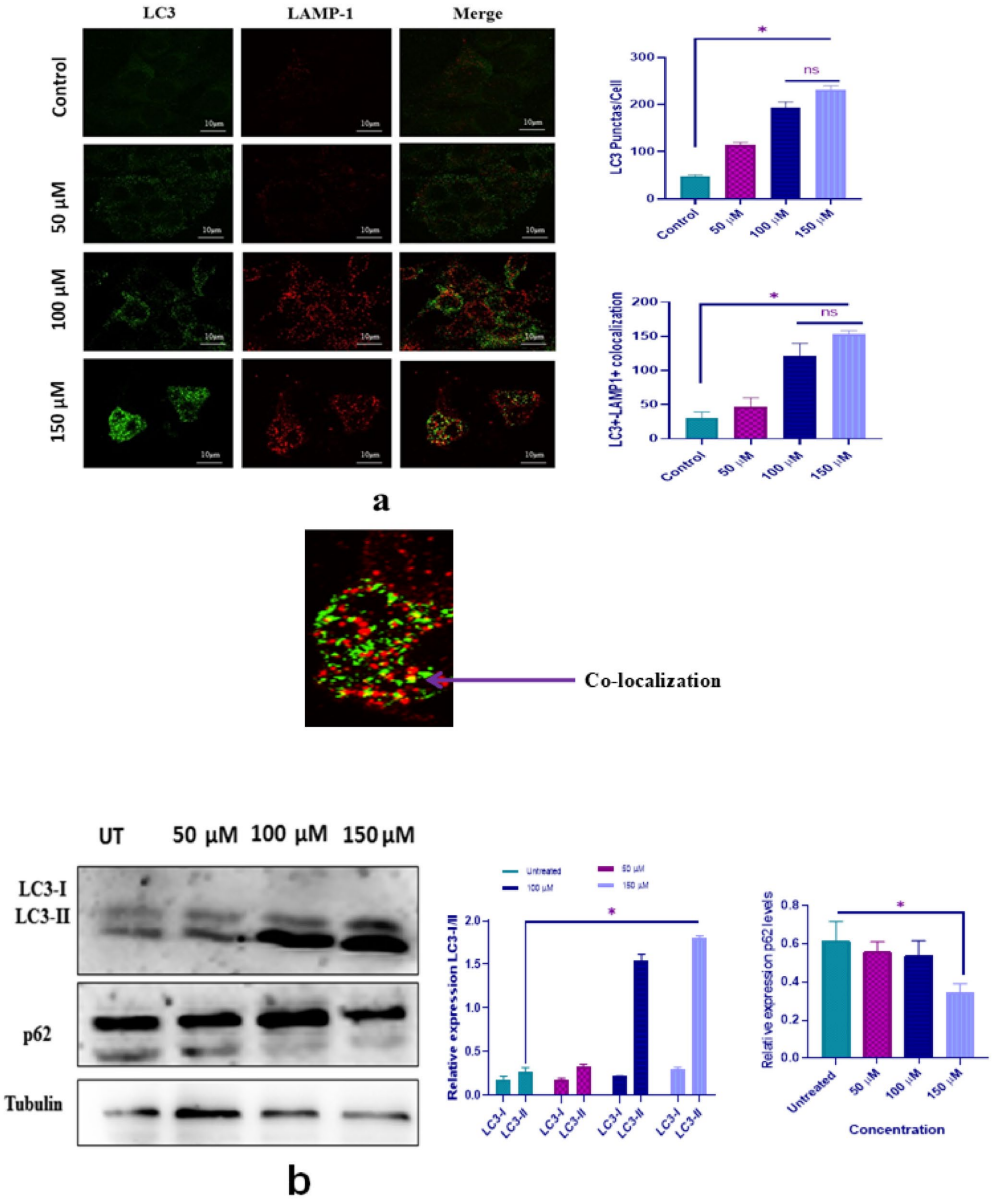
(a) Immunofluorescence images showing LC3‐II (green) and LAMP‐1 (red) puncta in MCF‐7 cells treated with increasing concentrations of naringenin (50, 100, and 150 μM) for 24 h. Merged images indicate increased LC3–LAMP1 co‐localization (yellow puncta), suggesting autophagosome–lysosome fusion. Quantification of LC3‐II puncta per cell and LC3‐II + LAMP1 co‐localiZation is shown in the bar graphs. Scale bars: 10 μm. (b) Western blot analysis of LC3‐I/LC3‐II and p62 expression, normalised to tubulin. Increased LC3‐II expression and decreased p62 levels indicate autophagy activation. The bar graphs quantify protein expression relative to untreated controls. Data represent mean ± SD from three independent experiments (*p < 0.05).

Figure 11 illustrates the increase in LC3‐II/LAMP‐1 puncta formation and the upregulation of LC3‐II expression along with p62 degradation in MCF‐7 cells treated with different concentrations of NAR compared to the control.

**FIGURE 12:**
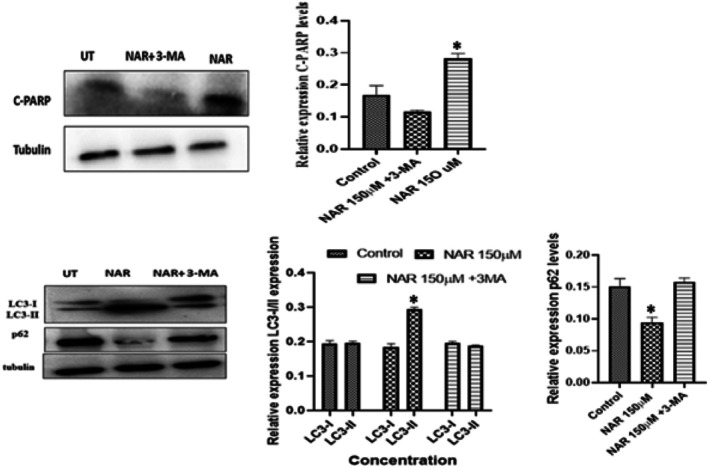
Naringenin promotes pro‐apoptotic autophagy in MCF‐7 Cells which is reversed by 3‐MA. (Top left) Western blot analysis of cleaved PARP (C‐PARP) expression in MCF‐7 cells treated with naringenin (150 μM) with or without 3‐MA (autophagy inhibitor). Tubulin was used as a loading control. (Top right) Quantification of C‐PARP levels, showing reduced apoptosis in the presence of 3‐MA. (Bottom left) Western blot analysis of LC3‐I/LC3‐II and p62 expression in the same conditions. (Bottom right) Quantification of LC3‐II and p62 levels, showing that NAR increases LC3‐II and reduces p62, while 3‐MA reverses these effects, confirming autophagy inhibition. Data represent mean ± SD from three independent experiments. *p < 0.05.

We further investigated whether NAR induced cell death occurred via apoptosis, or autophagy. For this, MCF‐7 cells were given NAR alone and in combination with 3‐MA which inhibits the initiation step in autophagy. The results revealed that upon combining NAR with 3‐MA, C‐PARP expression and LC3‐II expression were significantly decreased, thus displaying pro‐apoptotic autophagy as evidenced by the addition of 3‐MA shown in Figure 12.

## 4 Discussion

The present study evaluates the anticancer potential of NAR in breast cancer, demonstrating its ability to inhibit proliferation, induce apoptosis, and modulate autophagy in MCF‐7 cells. The results indicate that NAR exerts significant cytotoxic effects in a dose‐dependent manner, supporting its potential role in breast cancer therapy. Cell viability assays, including MTT and colony formation, confirmed that NAR significantly reduced MCF‐7 cell proliferation. The inhibitory effect on cell migration, as observed in the wound healing assay, suggests that NAR may suppress metastatic potential. These findings are consistent with those of Adam et al. who demonstrated that NAR inhibits cellular proliferation using assays such as MTT, colony formation, and wound healing [37]. Several other reports are also consistent with this study [24]. Cell cycle dysregulation is a hallmark of cancer progression, contributing to uncontrolled cell division and tumour growth [38]. In this study, NAR induced cell cycle arrest at the G0/G1 and S phases in a dose‐dependent manner, which is in accordance with the study conducted by Sultan et al., where they have shown that NAR arrests cell cycle at the G0/G1 phase in human epidermoid carcinoma cells [39]. In another study by Martínez‐Rodríguez et al., where they reported that NAR inhibits the cell cycle in the G0/G1 phase in cervical cancer spheroids [40]. Arrest at these phases suggests potential interactions with key regulatory proteins such as cyclins and cyclin‐dependent kinases, which should be explored in future studies.

ROS influence various cellular processes, including proliferation, apoptosis, and autophagy. An increase in ROS levels can promote oxidative stress, leading to DNA damage and activation of apoptotic pathways [41]. The results indicate that NAR treatment significantly elevated ROS generation, as evidenced by an increase in median fluorescence intensity (MFI). The results are inconsistent with the previous reports; Du et al. reported that NAR induces apoptosis due to increased generation of ROS [42]. In another study conducted by Lee et al. reported that NAR induces ROS generation, which leads to the induction of autophagy and apoptosis in human osteosarcoma cell lines [23]. This suggests that NAR‐induced apoptosis may be mediated through oxidative stress, which could be further investigated using ROS scavengers such as N‐acetylcysteine (NAC) to determine its role in cell death mechanisms. Apoptotic markers, including cleaved PARP (C‐PARP), chromatin condensation, and nuclear fragmentation, were evaluated to assess NAR‐induced cell death. Western blot analysis confirmed a significant increase in C‐PARP expression, a key indicator of apoptosis [43, 44]. This aligns with the findings of Song et al. who reported that NAR‐mediated increase in cleaved‐PARP contributes to apoptosis in human colon cancer cells [45].

DAPI staining further supported these findings by revealing nuclear condensation and fragmentation in treated cells. These observations align with previous studies highlighting the ability of flavonoids to activate apoptotic pathways in cancer cells. Given that many chemotherapeutic agents induce apoptosis through similar mechanisms, NAR may have potential as an adjunct to conventional therapies. The study also examined the role of NAR in autophagy induced cell death. Autophagy is a dynamic process that can either promote cell survival or contribute to autophagic cell death, depending on the cellular context. Immunofluorescence analysis demonstrated increased LC3 and LAMP‐1 puncta formation, indicating enhanced autophagosome‐lysosome fusion. Western blot analysis further confirmed increased LC3‐II expression and p62 degradation, suggesting activation of the autophagic pathway [46]. These results are consistent with Lee et al. who reported that NAR induces autophagy in osteosarcoma cells by increasing LC3‐II expression and P62 degradation [23].

Several studies have reported that autophagy can enhance or attenuate apoptosis in cancer cells [39].

Although the study provides strong evidence for the anticancer effects of NAR, some limitations should be addressed in future research. The experiments were conducted in vitro, and in vivo validation using breast cancer xenograft models would be necessary to confirm these findings in a physiological setting. Moreover, NAR's bioavailability and pharmacokinetics remain key factors that could influence its therapeutic potential. Investigating its efficacy in combination with standard chemotherapeutic agents could provide valuable insights into possible synergistic effects. In summary, the study demonstrates that NAR exerts antiproliferative effects, induces apoptosis through oxidative stress‐mediated mechanisms, and modulates autophagy in MCF‐7 cells. The evidence suggests that NAR‐induced autophagy functions in a pro‐apoptotic manner rather than as a cytoprotective response. Given its natural origin and minimal toxicity, NAR represents a potential candidate for breast cancer therapy. Further research focusing on its in vivo efficacy, molecular targets, and combination with existing treatments could enhance its translational potential in cancer therapeutics. Therefore, we investigated whether NAR induced cell death via apoptosis or autophagy. Our results revealed that NAR displayed pro‐apoptotic autophagy, as evidenced by the addition of 3‐MA, an autophagy inhibitor, resulting in reduced levels of C‐PARP and LC3‐II, suggesting that NAR‐induced autophagy facilitates apoptosis rather than acting as a protective mechanism. These findings are consistent with previous studies indicating that autophagy can either enhance or attenuate apoptosis depending on the molecular context. Further investigation into the upstream regulators of NAR‐induced autophagy, such as the PI3K/Akt/mTOR and AMPK pathways, could provide additional insights into its mechanism of action.

### 4.1 Strengths and Limitations

This study provides a comprehensive evaluation of NAR's anticancer effects in breast cancer, employing a diverse set of assays to assess its impact on cell proliferation, migration, apoptosis, and autophagy. The integration of MTT and colony formation assays for cell viability, wound healing assays for migration, and flow cytometry‐based apoptosis detection (Annexin V/PI and ROS generation assays) ensures a robust experimental approach. Western blotting and immunofluorescence further strengthen the findings by providing molecular insights into apoptotic and autophagic pathways. A notable strength is the dose‐dependent response observed across multiple assays, confirming the cytotoxic effects of NAR at increasing concentrations. Additionally, the use of 3‐MA as an autophagy inhibitor allowed the differentiation between apoptosis‐driven and autophagy‐driven cell death, adding mechanistic depth to the study. Despite these strengths, the study has certain limitations. The in vitro nature of the experiments limits the direct translation of findings to clinical applications. In vivo validation using breast cancer xenograft models would be necessary to confirm the therapeutic potential of NAR under physiological conditions. Another limitation is the use of a single breast cancer cell line (MCF‐7), representing ER‐positive luminal‐type breast cancer. Given the heterogeneity of breast cancer, future studies should evaluate NAR's effects on triple‐negative (MDA‐MB‐231) and HER2‐positive (SK‐BR‐3) cell lines to determine whether its cytotoxic effects are hormone receptor‐dependent. Additionally, the study primarily focuses on protein expression analysis of apoptosis and autophagy markers (C‐PARP, LC3‐II, and p62) but does not explore upstream regulatory pathways such as PI3K/Akt/mTOR, AMPK, or MAPK. Investigating these pathways could provide further insight into NAR's mechanism of action. While ROS generation was assessed, the study does not establish whether ROS‐induced apoptosis is caspase‐dependent or independent. The inclusion of ROS scavengers, such as N‐acetylcysteine (NAC) or Trolox, could clarify this mechanism. Furthermore, although cisplatin was used as a control in the migration assay, the study does not compare NAR's efficacy against standard chemotherapeutic agents such as paclitaxel or doxorubicin. Such comparisons would provide greater clinical relevance and highlight NAR's potential as a monotherapy or adjunct treatment in breast cancer.

## 5 Conclusion

This study provides comprehensive evidence that NAR exhibits potent anticancer effects in MCF‐7 breast cancer cells through its ability to inhibit proliferation, suppress migration, induce cell cycle arrest, and promote pro‐apoptotic autophagy. The findings demonstrate that NAR triggers cell cycle arrest at the G0/G1 and S phases, enhances ROS‐mediated apoptosis, and promotes autophagic activity by increasing LC3‐II expression and p62 degradation. Additionally, the use of 3‐MA as an autophagy inhibitor confirms that NAR‐induced apoptosis is partially mediated by autophagy activation. These results contribute to the growing body of research supporting flavonoids as promising anticancer agents, reinforcing NAR's potential as a therapeutic compound for breast cancer. However, further studies are necessary to explore its effects in vivo models, investigate its molecular mechanisms in greater detail, and evaluate its potential in combination therapies with standard chemotherapeutic agents.

## Author Contributions

**Suhail Ahmad Mir:** conceptualization (lead), data curation (lead), investigation (lead), methodology (lead), writing – original draft (lead), writing – review and editing (lead). **Basharat Ahmad Bhat:** data curation (supporting), formal analysis (supporting), validation (supporting). **Priti S. Shenoy:** investigation (supporting), methodology (supporting). **Laraibah Hamid:** formal analysis (equal), writing – original draft (equal), writing – review and editing (equal). **Nasir Nisar:** data curation (supporting), validation (supporting), writing – review and editing (supporting). **Ashraf Dar:** conceptualization (equal), resources (equal), supervision (equal), validation (equal). **Pritha Ray:** resources (equal), supervision (equal). **Ghulam Nabi Bader:** conceptualization (equal), resources (equal), supervision (equal), validation (equal).

## Ethics Statement

The authors have nothing to report.

## Consent

The authors have nothing to report.

## Conflicts of Interest

The authors declare no conflicts of interest.

## Data Availability

The data that support the findings of this study are available from the corresponding authors upon reasonable request.

## References

[1] H. Sung , J. Ferlay , R. L. Siegel , et al., “Global Cancer Statistics 2020: GLOBOCAN Estimates of Incidence and Mortality Worldwide for 36 Cancers in 185 Countries,” CA: A Cancer Journal for Clinicians 71 (2021): 209–249, 10.3322/caac.21660. 33538338

[2] S. A. Mir , L. Hamid , G. N. Bader , et al., “Role of Nanotechnology in Overcoming the Multidrug Resistance in Cancer Therapy: A Review,” Molecules 27 (2022): 6608, 10.3390/molecules27196608. 36235145 PMC9571152

[3] E. Morgan , M. Arnold , A. Gini , et al., “Global Burden of Colorectal Cancer in 2020 and 2040: Incidence and Mortality Estimates From GLOBOCAN,” Gut 72 (2023): 338–344, 10.1136/gutjnl-2022-327736. 36604116

[4] E. I. Obeagu and G. U. Obeagu , “Breast Cancer: A Review of Risk Factors and Diagnosis,” Medicine (Baltimore) 103 (2024): e36905, 10.1097/MD.0000000000036905. 38241592 PMC10798762

[5] K. M. Cuthrell , K. Morton Cuthrell , and N. Tzenios , “Breast Cancer: Updated and Deep Insights,” International Research Journal of Oncology 6 (2023): 104–118.

[6] S. Łukasiewicz , M. Czeczelewski , A. Forma , J. Baj , R. Sitarz , and A. Stanisławek , “Breast Cancer—Epidemiology, Risk Factors, Classification, Prognostic Markers, and Current Treatment Strategies—An Updated Review,” Cancers 13 (2021): 4287, 10.3390/cancers13174287. 34503097 PMC8428369

[7] A. Ansari , M. Agarwal , V. K. Singh , K. Nutan , and T. J. Deori , “Breast Cancer Literacy: Status of Peripheral Health Workers in Lucknow,” Asian Pacific Journal of Cancer Care 8 (2025): 287–294, 10.31557/APJCC.2023.8.2.287.

[8] O. Amato , V. Guarneri , and F. Girardi , “Epidemiology Trends and Progress in Breast Cancer Survival: Earlier Diagnosis, New Therapeutics,” Current Opinion in Oncology 35 (2023): 612–619, 10.1097/CCO.0000000000000991. 37681462 PMC10566595

[9] M. A. Mir and M. A. Mir , “Current Treatment Approaches to Breast Cancer,” in Therapeutic Potential of Cell Cycle Kinases in Breast Cancer (Springer, 2023), 10.1007/978-981-19-8911-7.

[10] A. Burguin , C. Diorio , and F. Durocher , “Breast Cancer Treatments: Updates and New Challenges,” Journal of Personalized Medicine 11 (2021): 808, 10.3390/jpm11080808. 34442452 PMC8399130

[11] K. P. Trayes and S. E. H. Cokenakes , “Breast Cancer Treatment,” American Family Physician 104 (2021): 171–178. 34383430

[12] M. Nedeljković and A. Damjanović , “Mechanisms of Chemotherapy Resistance in Triple‐Negative Breast Cancer—How we Can Rise to the Challenge,” Cells 8 (2019): 957, 10.3390/cells8090957. 31443516 PMC6770896

[13] P. d. F. Lainetti , A. F. Leis‐Filho , R. Laufer‐Amorim , A. Battazza , and C. E. Fonseca‐Alves , “Mechanisms of Resistance to Chemotherapy in Breast Cancer and Possible Targets in Drug Delivery Systems,” Pharmaceutics 12 (2020): 1193, 10.3390/pharmaceutics12121193. 33316872 PMC7763855

[14] A. Catalano , D. Iacopetta , J. Ceramella , et al., “Multidrug Resistance (MDR): A Widespread Phenomenon in Pharmacological Therapies,” Molecules 27 (2022): 616, 10.3390/molecules27030616. 35163878 PMC8839222

[15] A. Esmeeta , S. Adhikary , V. Dharshnaa , et al., “Plant‐Derived Bioactive Compounds in Colon Cancer Treatment: An Updated Review,” Biomedicine & Pharmacotherapy 153 (2022): 113384, 10.1016/j.biopha.2022.113384. 35820317

[16] S. Hashem , T. A. Ali , S. Akhtar , et al., “Targeting Cancer Signaling Pathways by Natural Products: Exploring Promising Anti‐Cancer Agents,” Biomedicine & Pharmacotherapy 150 (2022): 113054, 10.1016/j.biopha.2022.113054. 35658225

[17] K. R. Landis‐Piwowar and N. R. Iyer , “Cancer Chemoprevention: Current State of the Art,” Cancer Growth & Metastasis 7 (2014): CGM.S11288, 10.4137/CGM.S11288. PMC4064948 24987270

[18] L. Tariq , B. A. Bhat , S. S. Hamdani , and R. A. Mir , “Phytochemistry, Pharmacology and Toxicity of Medicinal Plants,” in Medicinal and Aromatic Plants (Springer International Publishing, 2021), 217–240, 10.1007/978-3-030-58975-2_8.

[19] S. A. Mir , A. Dar , L. Hamid , et al., “Flavonoids as Promising Molecules in the Cancer Therapy: An Insight,” Current Research in Pharmacology and Drug Discovery 6 (2024): 100167, 10.1016/j.crphar.2023.100167. 38144883 PMC10733705

[20] D. M. Kopustinskiene , V. Jakstas , A. Savickas , and J. Bernatoniene , “Flavonoids as Anticancer Agents,” Nutrients 12 (2020): 457, 10.3390/nu12020457. 32059369 PMC7071196

[21] S. Singh , A. Sharma , V. Monga , and R. Bhatia , “Compendium of Naringenin: Potential Sources, Analytical Aspects, Chemistry, Nutraceutical Potentials and Pharmacological Profile,” Critical Reviews in Food Science and Nutrition 63 (2023): 8868–8899, 10.1080/10408398.2022.2056726. 35357240

[22] B. B. Chandrika , M. Steephan , T. R. S. Kumar , A. Sabu , and M. Haridas , “Hesperetin and Naringenin Sensitize HER2 Positive Cancer Cells to Death by Serving as HER2 Tyrosine Kinase Inhibitors,” Life Sciences 160 (2016): 47–56, 10.1016/j.lfs.2016.07.007. 27449398

[23] C.‐W. Lee , C. C.‐Y. Huang , M.‐C. Chi , et al., “Naringenin Induces ROS‐Mediated ER Stress, Autophagy, and Apoptosis in Human Osteosarcoma Cell Lines,” Molecules 27 (2022): 373, 10.3390/molecules27020373. 35056691 PMC8781290

[24] X. Shi , X. Luo , T. Chen , et al., “Naringenin Inhibits Migration, Invasion, Induces Apoptosis in Human Lung Cancer Cells and Arrests Tumour Progression In Vitro,” Journal of Cellular and Molecular Medicine 25 (2021): 2563–2571, 10.1111/jcmm.16226. 33523599 PMC7933922

[25] H. Cheng , X. Jiang , Q. Zhang , et al., “Naringin Inhibits Colorectal Cancer Cell Growth by Repressing the PI3K/AKT/mTOR Signaling Pathway,” Experimental and Therapeutic Medicine 19 (2020): 3798–3804, 10.3892/etm.2020.8649. 32346444 PMC7185071

[26] R. Wang , J. Wang , T. Dong , J. Shen , X. Gao , and J. Zhou , “Naringenin Has a Chemoprotective Effect in MDA‐MB‐231 Breast Cancer Cells via Inhibition of Caspase‐3 and ‐9 Activities,” Oncology Letters 17, no. 1 (2018): 1217–1222, 10.3892/ol.2018.9704. 30655887 PMC6312985

[27] P. K. Ajji , K. Walder , and M. Puri , “Combination of Balsamin and Flavonoids Induce Apoptotic Effects in Liver and Breast Cancer Cells,” Frontiers in Pharmacology 11 (2020): 11, 10.3389/fphar.2020.574496. 33192517 PMC7655928

[28] X. Wang , C. C. Decker , L. Zechner , S. Krstin , and M. Wink , “In Vitro Wound Healing of Tumor Cells: Inhibition of Cell Migration by Selected Cytotoxic Alkaloids,” BMC Pharmacology and Toxicology 20 (2019): 4, 10.1186/s40360-018-0284-4. 30626448 PMC6327619

[29] P. Y. K. Yue , E. P. Y. Leung , N. K. Mak , and R. N. S. Wong , “A Simplified Method for Quantifying Cell Migration/Wound Healing in 96‐Well Plates,” SLAS Discovery 15 (2010): 427–433, 10.1177/1087057110361772. 20208035

[30] N. A. P. Franken , H. M. Rodermond , J. Stap , J. Haveman , and C. van Bree , “Clonogenic Assay of Cells in Vitro,” Nature Protocols 1 (2006): 2315–2319, 10.1038/nprot.2006.339. 17406473

[31] C. Xu , X. Huang , Y. Huang , et al., “Naringin Induces Apoptosis of Gastric Carcinoma Cells via Blocking the PI3K/AKT Pathway and Activating Pro‐Death Autophagy,” Molecular Medicine Reports 24 (2021): 772, 10.3892/mmr.2021.12412. 34490484 PMC8441985

[32] M. Y. Alfaifi , “Kanahia Laniflora Methanolic Extract Suppressed Proliferation of Human Non‐Small Cell Lung Cancer A549 Cells,” Asian Pacific Journal of Cancer Prevention 17 (2016): 4755–4759, 10.22034/APJCP.2016.17.10.4755. 27893208 PMC5454628

[33] D. Xie , P. Yuan , D. Wang , H. Jin , and H. Chen , “Effects of Naringin on the Expression of miR‐19b and Cell Apoptosis in Human Hepatocellular Carcinoma,” Oncology Letters 14 (2017): 1455–1459, 10.3892/ol.2017.6278. 28789364 PMC5529873

[34] M. Zhang , J. Lai , Q. Wu , et al., “Naringenin Induces HepG2 Cell Apoptosis via ROS‐Mediated JAK‐2/STAT‐3 Signaling Pathways,” Molecules 28 (2023): 4506, 10.3390/molecules28114506. 37298981 PMC10254813

[35] H. Xiong , Z. Chen , B. Lin , et al., “Naringenin Regulates FKBP4/NR3C1/NRF2 Axis in Autophagy and Proliferation of Breast Cancer and Differentiation and Maturation of Dendritic Cell,” Frontiers in Immunology 12 (2022): 12, 10.3389/fimmu.2021.745111. PMC8786807 35087512

[36] A. U. Ahsan , V. L. Sharma , A. Wani , and M. Chopra , “Naringenin Upregulates AMPK‐Mediated Autophagy to Rescue Neuronal Cells From β‐Amyloid (1–42) Evoked Neurotoxicity,” Molecular Neurobiology 57 (2020): 3589–3602, 10.1007/s12035-020-01969-4. 32542594

[37] A. Hermawan , M. Ikawati , R. I. Jenie , et al., “Identification of Potential Therapeutic Target of Naringenin in Breast Cancer Stem Cells Inhibition by Bioinformatics and in Vitro Studies,” Saudi Pharmaceutical Journal 29 (2021): 12–26, 10.1016/j.jsps.2020.12.002. 33603536 PMC7873751

[38] Z. Zhong , X. Chen , W. Tan , et al., “Germacrone Inhibits the Proliferation of Breast Cancer Cell Lines by Inducing Cell Cycle Arrest and Promoting Apoptosis,” European Journal of Pharmacology 667 (2011): 50–55, 10.1016/j.ejphar.2011.03.041. 21497161

[39] M. S. Ahamad , S. Siddiqui , A. Jafri , S. Ahmad , M. Afzal , and M. Arshad , “Induction of Apoptosis and Antiproliferative Activity of Naringenin in Human Epidermoid Carcinoma Cell Through ROS Generation and Cell Cycle Arrest,” PLoS One 9 (2014): e110003, 10.1371/journal.pone.0110003. 25330158 PMC4199682

[40] O. P. Martínez‐Rodríguez , A. González‐Torres , L. M. Álvarez‐Salas , et al., “Effect of Naringenin and Its Combination With Cisplatin in Cell Death, Proliferation and Invasion of Cervical Cancer Spheroids,” RSC Advances 11 (2021): 129–141, 10.1039/D0RA07309A. PMC8690252 35423031

[41] H. J. Park , Y. J. Choi , J. H. Lee , and M. J. Nam , “Naringenin Causes ASK1‐Induced Apoptosis via Reactive Oxygen Species in Human Pancreatic Cancer Cells,” Food and Chemical Toxicology 99 (2017): 1–8, 10.1016/j.fct.2016.11.008. 27838343

[42] Y. Du , J. Lai , J. Su , et al., “Naringenin‐Induced Oral Cancer Cell Apoptosis via ROS‐Mediated Bid and Bcl‐Xl Signaling Pathway,” Current Cancer Drug Targets 24 (2024): 668–679, 10.2174/0115680096267430231023091521. 38178673

[43] A. Saraste , “Morphologic and Biochemical Hallmarks of Apoptosis,” Cardiovascular Research 45 (2000): 528–537, 10.1016/S0008-6363(99)00384-3. 10728374

[44] S. Gobeil , C. C. Boucher , D. Nadeau , and G. G. Poirier , “Characterization of the Necrotic Cleavage of Poly(ADP‐Ribose) Polymerase (PARP‐1): Implication of Lysosomal Proteases,” Cell Death and Differentiation 8 (2001): 588–594, 10.1038/sj.cdd.4400851. 11536009

[45] H. M. Song , G. H. Park , H. J. Eo , and J. B. Jeong , “Naringenin‐Mediated ATF3 Expression Contributes to Apoptosis in Human Colon Cancer,” Biomolecules & Therapeutics 24 (2016): 140–146, 10.4062/biomolther.2015.109. 26797111 PMC4774494

[46] S. Raha , S. Yumnam , G. E. Hong , et al., “Naringin Induces Autophagy‐Mediated Growth Inhibition by Downregulating the PI3K/Akt/mTOR Cascade via Activation of MAPK Pathways in AGS Cancer Cells,” International Journal of Oncology 47 (2015): 1061–1069, 10.3892/ijo.2015.3095. 26201693

